# Upgrading the fixed-time artificial insemination (FTAI) protocol in Romanian buffaloes

**DOI:** 10.3389/fvets.2023.1265060

**Published:** 2023-10-27

**Authors:** Stefan Gregore Ciornei, Petru Roşca

**Affiliations:** Department of Clinical Sciences, Faculty of Veterinary Medicine, Iasi University of Life Sciences, Iasi, Romania

**Keywords:** estrus inductions, Romanian buffalo, FTAI, OvSynch, sperm

## Abstract

The present study describes the challenges of assisted reproduction in Romanian buffaloes while increasing the efficacy of artificial insemination by choosing the most suitable method. The modified fixed-time artificial insemination (FTAI) protocol with sexed semen was used to increase the conception rate. This study included a total of 80 buffalo heifers that received ovarian stimulation using the OvSynch protocol. Two groups (*n* = 40), namely, a control group, in which the classic FTAI method was performed, and an experimental group, in which deep intrauterine AI was performed in cows that had developed a dominant follicle (US+UcFTAI), were randomly selected. The conception rate (CR) was 63.6% in the experimental group, which was statistically higher (*P* < 0.05) than the control group (30%). The ultrasound examination indicated that, using the OvSynch protocol, 82.5% (33 out of 40) of buffaloes developed a dominant follicle (DF) while the distribution between the warm and cold seasons was 75 and 90%, respectively. The CR was 60% during the hot season and 66.6% during the cold season. At calving, 92.5% female fetuses were born. The improved FTAI method in this study enhanced the results by reducing the waste of sexed semen and maximizing the response to OvSynch, making it a recommendation for practitioners. This study presents preliminary results and highlights that genetic progress is difficult to achieve. A systematic approach is needed in order to choose the most suitable biotechnological method for each farm.

## 1. Introduction

Various animal husbandry systems are currently used in most buffalo breeding countries, such as India (1), Brazil (2), Italy (3), and China (4, 5), within smaller or larger farms. As the number of local buffaloes in Romania is constantly decreasing, the application of current reproductive biotechnologies in these breeds remains limited (6, 7). Several European countries with a strong tradition in milk buffalo production heavily rely on AI. Nevertheless, many farms continue to use bulls and combine AI with natural breeding methods (3).

The Romanian buffalo is characterized by seasonal reproductive activity, with over 50.8% spontaneous estrus occurring during the spring season. They typically reach sexual maturity at a later stage, after 22 months, and have a calving interval between 443 and 547 days. Estrus lasts only for a short duration of time, i.e., 8–12 h, and is weakly expressed clinically, with small dominant and ovulatory follicles measuring 9–1.2 cm and poorly developed CL of 1 cm. Although the ovaries are small in size, (measuring 24.3 mm in length, 18.25 mm in width, 13.15 mm in thickness, and weighing 4.53 g), they have a very high reactive activity. The conception rate achieved through AI varies between 26 and 30% (6, 8).

The current research study indicates promising prospects for combining assisted reproduction and genomic evaluation, which can be used successfully for populations with limited sizes (9). Deep uterine AI offers the advantage of depositing semen closer to the utero-tubal junction (10) while allowing for the use of a lower dose and sexed semen (11). Utilizing ipsilateral ultrasound guidance toward the ovary with DF (8) leads to an increase in the conception rate.

In the past decade, assisted reproduction in Romanian buffaloes has yielded unsatisfactory outcomes; however, research development and optimization of reproductive biotechniques have begun to produce promising results (6–8, 12, 13).

Anestrus in buffaloes poses a costly challenge and a reproductive problem, resulting in a low conception rate. Researchers worldwide have employed various estrus induction protocols utilizing different hormones to address anestrus, with varying success rates (14–18).

Poor endocrine status of buffaloes (19), lower number of follicles (20), high incidence of silent estrus, long calving interval (12), weak palpable ovarian structures on transrectal examination (18, 21), seasonality of reproduction and poor fertility (3), and high incidence of follicular atresia (22) are some of the limiting factors that probably resulted in moderate responses of buffalo cows to reproductive biotechnologies (18). Currently, there are several limiting factors affecting the implementation of AI in buffaloes. Therefore, the use of hormonal protocols associated with FTAI makes reproduction in buffaloes more advantageous and practical (18, 21, 23). Furthermore, the small number of genomically tested buffalo bulls, compared to cattle, and the low conception rates obtained in AI programs discouraged buffalo farmers from adopting this technique (6). Although reproduction management protocols are similar to those of cows, they have a limited application due to low estrus expression and poor detection rates, a variable duration of estrus, and difficulty in predicting the time of ovulation (12).

Since its development, ovulation synchronization (OvSynch) and timed artificial insemination (TAI) programs (OvSynch+TAI) have significantly reduced the time and costs associated with reproduction (24).

During the breeding season, using the OvSynch protocol together with FTAI for 16–20 h, after the second GnRH injection, yielded an acceptable CR (35%-56%) in buffaloes although lower conception rates (CR) have also been reported (25, 26). Subsequently, the model was adopted and tested on river buffaloes in different seasons (19, 21) and implemented to a limited extent in swamp buffaloes as well (22, 27).

These data could be beneficial in improving and establishing the most suitable reproductive model for future studies and for the efficiency of reproductive biotechnologies in the buffalo industry.

This study aims to describe the challenges of assisted reproduction in buffaloes, including low conception rates after implementing AI, susceptibility to seasonality, and difficulties in estrus detection. These challenges are primarily attributed to unique characteristics of the estrous cycle in Romanian buffaloes, where signs of estrus are weakly expressed clinically, accompanied by a short estrus duration, small dominant and pre-ovulatory follicles, and underdeveloped CL. Moreover, an improved FTAI method using sexed semen was tested to increase the conception rate.

## 2. Materials and methods

### 2.1. Buffalo cows

The experiment was performed on a total of 80 primiparous buffalo cows, specifically of the Romanian Indigenous Buffalo (RIB) variety, belonging to the Mediterranean breed. Our goal was to form a nucleus of females with increased genetic potential.

Young buffalo heifers were selected based on their age (over 22 months), weight (over 250 kg), and a body condition score (BCS) of around 3. In terms of sexual cyclicity, only cattle that had passed the onset of puberty and had active physiological structures on the ovaries were selected. The ovaries and uterus showed no signs of pathology, and the shape and size of the ovaries were within the normal range, measuring at least 2 cm in width and displaying physiological structures.

**Figure 1:**
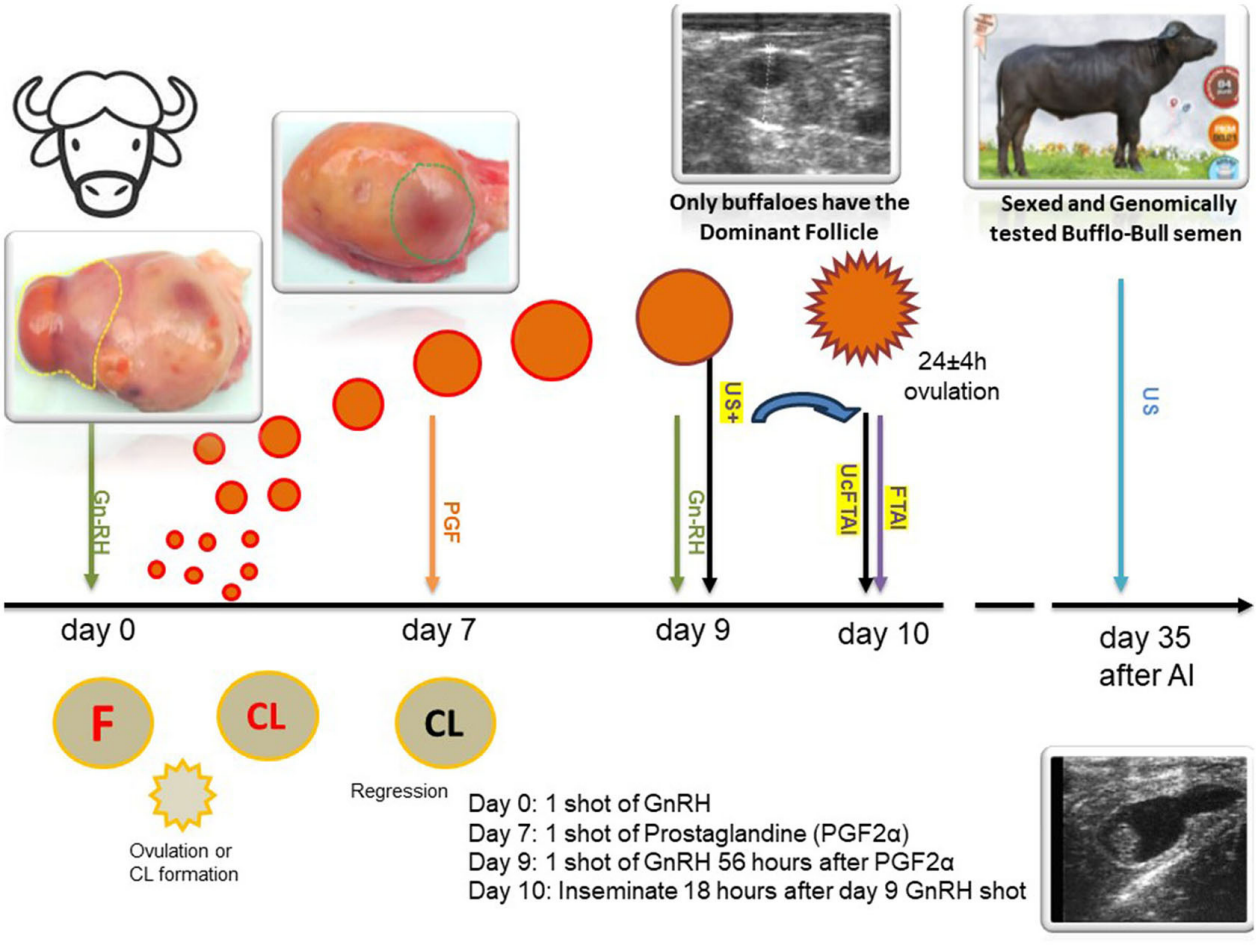
Scheme of the US+UcFTAI and FTAI method with semen sexed according to the OvSynch protocol in Romanian buffalo (CIB).

The group containing 80 female buffaloes was subjected to a thorough gynecological and general examination (see Figure 1). They were then divided into two groups: a control group and an experimental group, containing equal number of buffaloes in each group. Each female buffalo was randomly selected from either the control or experimental group (*n* = 40 FTAI/US+UcFTAI). Then, AI was performed using sexed semen from two fertile bulls during both the warm and cold seasons. It is worth noting that, locally, the breeding season aligns with the cold months (cold season).

### 2.2. Buffalo bull semen

Two different bulls, tested genomically and using sexed semen, from Associatzione Nazionale Allevatori Specie Bufalina (Italy) Oro Mediterraneo and Aton del Parco, were used. The semen was purchased, transported, and stored in optimal conditions by a local importer, in straws containing 2 million X-chromosome-bearing sperm.

### 2.3. Care management

All biosecurity, nutrition, welfare, maintenance, vaccination, and deworming conditions were ensured. For ensuring that their living conditions were comfortable, the animals had thermic comfort, by preventing extreme heat or cold. This allowed for continuous reproductive activity, without the usual seasonal changes.

### 2.4. Ultrasound monitoring

Ovarian dynamics and follicular evolution of buffalo cows during the estrous synchronization treatment were monitored using transrectal ultrasonography with a Honda HS-1600V^®^ ultrasound scanner from Japan equipped with a 5–7.5 MHz and Doppler transducer (8). Transrectal genital ultrasound examination was performed at three crucial time points: before assembling the experimental and control groups to exclude those with uterine/ovarian pathology, ensuring that only normally cycling cows were included; 18 h before the scheduled time for AI to select heifers that had a DF on the ovary (US+UcFTAI), and 35 days after AI for pregnancy diagnosis and calculation of the conception rate (see Figure 1).

### 2.5. Hormone treatment management

The groups, control and experimental groups, were formed after a thorough gynecological and general examination. Subsequently, the OvSynch (GnRH-PGF-GnRH) protocol was initiated. According to the treatment scheme, buffaloes received IM injections on day 0 and 9 with a dosage of 0.01 mg buserelin acetate (Receptal^®^, MDS, The Netherlands). On the seventh day, they received a PGF2alpha synthetic analog, that is, cloprostenol 500 μg (Estrumate^®^, MDS, The Netherlands).

### 2.6. Artificial insemination and time management (FTAI/US+UcFTAI)

The control group was artificially inseminated on day 10 without the need to detect clinical signs of estrus, following the classic FTAI method. In the experimental group (US+UcFTAI), to prevent waste, only buffaloes with a dominant follicle (DF) measuring at least 0.9 mm on the ovary were inseminated on day 9 (27). The AI method employed was recto-vaginal, with the female buffaloes inseminated once, 18 h after the second GnRH injection (FT-fixed times). Sexed semen was deposited only in the uterine horn ipsilateral to the ovary carrying the dominant follicle (deep Uc-unicornual). The insemination technique followed the classical Anglo-Saxon method, by transrectally locating the cervix and transcervical passage of the Cassou insemination gun. A plastic protective sheet was also used.

### 2.7. Statistical analyses

Data of conception rates, estrus, and ovulation across the groups were compared through the chi-squared test, and the value of *p* of < 0.05 was considered statistically significant. Statistical analysis was performed using Prism version 8 (GraphPad software 5.0. La Jolla, CA, USA, www.graphpad.com, accessed on 28 August 2023).

## 3. Results

The experimental group, using the modified OvSynch protocol, US+UcFTAI, achieved a CR of 63.6% (21 out of 33) with a *p-*value of 0.00405. In the control group, all females were inseminated using FTAI protocol, resulting in a conception rate of 30% (12 out of 40) with differences between the warm and cold seasons (25 vs. 35%) (*P* > 0.05) and between Oro and Aton bulls (35 vs. 25%) (*p* > 0.05). At parturition, 91.6% females were obtained (Table 1).

**Table 1:** Conception rate in Romanian Indigenous Buffalo (RIB) according to the FTAI and ES+UcFTAI protocol.

**Semen**	**FTAI control** ^*^		**US** + Uc FTAI tested^*^		**Conception rate (%CR)** ^*^		**Calving (%)**
	**W.S**	**C.S**	**W.S**	**C.S**	**W.S**	**C.S**	**Female fetus**
Oro group	-	-	8/10 (80%)	9/10 (90%)	4/8 (50%)^e^	6/9 (66.6%)	10/10 (100%)
			17/20 (85%)		10/17 (58.8%)		
Aton group	-	-	7/10 (70%)	9/10 (90%)	5/7 (71.1%) ^f^	6/9 (66.6%)	10/11 (90.9%)
			16/20 (80%)		11/16 (68.7%)		
Total experimental group	-	-	15/20 (75%)	18/20 (90%)	9/15 (60%) ^g^	12/18 (66.6%) ^h^	20/21 (95.2%)
			33/40 (82.5%)^c^		21/33 (63.6%)^a^		
Oro group	10/20 (50%)	10/20 (50%)	-	-	3/10 (30%)	4/10 (40%)	6/7 (85.7%)
	20/20 (100%)				7/20 (35%)		
Aton group	10/20 (50%)	10/20 (50%)	-	-	2/10 (20%)	3/10 (30%)	5/5 (100%)
	20/20 (100%)				5/20 (25%)		
Total control group	20/40 (50%)	20/40 (50%)	-	-	5/20^i^ (25%)	7/20^j^ (35%)	11/12 (91.6%)
	40/40 (100%)^d^				12/40 (30%)^b^		

^*^Different superscripts^(a, b)^ represent significant differences (P < 0.05) within the same parameters (a/b p = 0.0040).

Different superscripts^(c, d)^ represent insignificant differences (P > 0.05) within the same parameters (c/d p = 0.056, e/f = 0.039, g/h = 0.691, i/j = 0.490).

w.s., warm season; c.s., cold season.

The statistical difference between the females inseminated in the control group and the experimental group that developed a DF was inconclusive with a *p*-value of 0.056 (>0.05). Before insemination, an ultrasound examination was performed. In the experimental group, 33 out of 40 buffaloes (82.5%) had a new dominant follicle of at least 0.9 cm in diameter in one of the ovaries. It is noteworthy to mention that 82.5% of females had a complete response and were considered to show a positive reaction to hormonal estrus induction, with or without behavioral clinical signs. These females were inseminated with semen from two buffalo bulls, with 85% from Oro's group and 80% from Aton's (see Table 1). Regarding seasonal distribution, the dominant follicle was detected in 75% (15 out of 20) of the buffaloes in the warm season and 90% (18 out of 20) during the cold season. The total conception rate was 63.6% (21 out of 33), which is statistically higher (*p* < 0.05) than the control group. The distribution between the two seasons (warm/cold) was 60% (9 out of 15) compared to 66.6% (12 out of 18), which was statistically insignificant (*p* > 0.05). By categories of bulls, the percentages were also similar, with 60% in Oro's group (9 out of 15) and 66.6% in Aton's (12 out of 18), (*p* < 0.05). A difference in fertility between bulls was only observed during the summer season, with 71.7% (5/7) in Aton's group and 50% (4/8) in Oro's group, but it was statistically insignificant (*p* > 0.05).

Regarding the sex ratio, after calving, 92.5% (20 out of 21) of female fetuses were obtained, and only in Aton's group, one male was born.

## 4. Discussion

As the number of buffaloes in Romania has been decreasing during the last few decades, it has become challenging to form homogeneous buffalo groups from the local variety (7). The seasonal buffalo anestrous in countries north of the equator has been attributed to factors such as thermic stress (India, Pakistan, and Egypt), low environmental humidity (Venezuela), or photoperiodicity (Italy and European Countries) (18). In Romania, the breeding season for the Romanian Indigenous Buffalo is considered to occur during the cold season (autumn–winter) (6, 12).

Some veterinary drugs, such as progestogens, gonadotropin-releasing hormone (GnRH), prostaglandin F2alpha (PGF2α), and eCG, have been used with different schemes, such as OvSynch, to control estrus (13) in buffaloes. However, the results have been variable and inconclusive (26, 27). Therefore, there is a need to continue the research efforts aimed at innovation in this field.

According to the literature, ovulation induced in heifers following the GnRH–PGF2α protocol can be anticipated within approximately 28 h (6, 21). The authors recommend the administration of GnRH at the end of the growth phase or at the beginning of the static phase of the dominant follicle, with GnRH administered within 72, 48, or 24 h after PGF2α (28).

Pursley et al. (29) were among the first to use a protocol for estrus synchronization and ovulation in cows with fixed-time insemination (19). Since then, various protocols based on OvSynch have emerged to improve CR (13, 21, 30). These protocols have also been applied to buffalo cows but yielded lower results compared to cows (26, 27). Recently, it was stated that CR varies between 30 and 60% (3, 4), depending on the season, treatment, management, or age (19), with rates ranging from 26 to 80%. The double OvSynch protocol, combined with ultrasound confirmation of pregnancy, was shown to improve the CR, in Italian farms, for constant milk production (3).

Genetic enhancement and the acquisition of individuals with superior production quality (18) can be achieved over several generations by implementing the classic breeding protocols and selecting only animals with validated productivity for breeding. Given that the buffalo population is small and the production value is low, it is necessary to use modern and current reproductive biotechnologies, such as artificial insemination (AI), as a measure of improvement and genetic progress in the buffalo herd. The methods and techniques are, and must be, in continuous development, with only those suitable for each farmer, species, and breed being employed (8, 24). It is noteworthy that AI is practiced very infrequently in Europe, with only 5% of buffaloes in Italy, and merely 0.1% in Romania (31).

Considering the physiological characteristics described above, regarding follicular development, the occurrence of a DF and ovulation following OvSynch and recognizing that not all females develop and ovulate a follicle, there arose a need to control the development of DFs through ultrasound examination before FTAI. The conception rate obtained was higher (63.6%) in this experimental group (US+UcFTAI). This increase was possible for two reasons: On the one hand, due to the fact that sexed semen was placed closer to the fertilization site, and on the other hand, due to the ultrasound selection of cows with highest likelihood to ovulate (present DF).

The results of this study (with CR of 63.6%) surpass other publications that only used the OvSynch protocol. These other studies reported conception rates of 48.8% (27), 56.5% (29) during the mating season, 56.7% (32) in multiparous females, and 6.9% (27) during the non-mating season. Other studies on CR after stimulation with OvSynch has shown variation between 36.0 and 42.55% when used during the breeding season with conventional semen (18, 30).

Studies published by Paul and Prakash, using the OvSynch protocol in non-lactating Murah buffaloes, reported an ovulation of 90% in 23.3 h, but the CR was 33.3% at FTAI (12 and 24 h after OvSynch) and 30.7% in spontaneous estrus (after 12 h of OvSynch-induced estrus) (33).

Moreover, by implementing the US+UcFTAI modified protocol in both the cold and warm seasons, a favorable ovarian reaction was observed in 82.5% of buffaloes. This study demonstrated consistent CR results between the warm and cold seasons, and because the maintenance and comfort conditions were optimal, the adverse effects of heat and frost, as well as ovarian inhibition, which are specific to buffaloes, were avoided.

## 5. Conclusion

Improvement of the FTAI protocol by incorporating ultrasound scanning and intracornual AI was performed to reduce waste and maximize the effectiveness of the OvSynch hormone therapy. The efficiency of the ovarian response was achieved through a careful gynecological selection of female heifers and bio-stimulation with bulls. It is recommended to conduct an ultrasound examination 18 h before FTAI, with the latter being ipsilateral and deeply intracornual. This approach is applied exclusively to buffaloes with DF larger than 0.9 cm. Thus, by implementing the upgraded protocol of UcFTAI with sexed semen, the objective of increasing the conception rate in buffalo populations with limited numbers becomes achievable and can potentially be expanded to a larger scale.

## Data availability statement

The original contributions presented in the study are included in the article/supplementary material, further inquiries can be directed to the corresponding author.

## Ethics statement

The animal study was approved by the Iasi University of Life Sciences (IULS), Faculty of Veterinary Medicine, Bioethics Committee following the EU 2010/63 and National Directives Ord. 28/31–08–2011 and National Law 206/2004. The study was conducted in accordance with the local legislation and institutional requirements.

## Author contributions

SC: Conceptualization, Data curation, Formal analysis, Investigation, Software, Writing—original draft, Writing—review and editing. PR: Resources, Supervision, Validation, Writing—review and editing.
